# Screening families after sudden cardiac death in the young: do we ever stop?

**DOI:** 10.1093/europace/euaf141

**Published:** 2025-07-16

**Authors:** Christopher Semsarian, Caroline Medi

**Affiliations:** Genetic Heart Clinic, Sydney Heart Health Centre, Lindfield, Sydney, NSW 2070, Australia; Department of Cardiology, Royal Prince Alfred Hospital, Camperdown, Sydney, NSW 2050, Australia

**This editorial refers to ‘Diagnostic yield in families to sudden cardiac death victims—a 10-year follow-up study’ by C.L. Grønholdt *et al*., https://doi.org/10.1093/europace/euaf119.**

Sudden cardiac death (SCD) is a tragic complication in a number of cardiovascular diseases. In those aged over 35 years, coronary artery disease and myocardial infarction are the most common causes of SCD. In the young aged <35 years, inherited cardiomyopathies such as arrhythmogenic cardiomyopathy (ACM) and hypertrophic cardiomyopathy (HCM), and inherited arrhythmia disorders such as long QT syndrome (LQTS) and Brugada syndrome (BrS), collectively account for over 50% of young SCD.^1–3^ In up to 40% of young people who die suddenly, no cause of death is identified after a comprehensive post-mortem evaluation.^1^ Given the significant proportion of SCD in the young that may be due to an inherited cause, clinical screening, as well as if available, proband genetic testing, are important steps in identifying other relatives of the decedent who may have the same disease and therefore be at risk of disease complications including arrhythmias and SCD.

To date, relatively small follow-up studies in SCD families have highlighted the importance of clinical and genetic screening in the family. Most of these studies have reported on the diagnosis of inherited heart disease in a relative during the first round of clinical screening following the young SCD.^1,4,5^ But a highly relevant and important question remains, i.e. in those relatives who are clinically normal at first screening, how frequently and for how long should these at-risk family members be clinically screened.

In this issue of *Europace*, Grønholdt *et al.*^6^ present one of the largest and longest follow-up investigations to date assessing the diagnostic yield of inherited heart disease in families affected by SCD. This retrospective, single-centre study sought to determine the 10-year diagnostic yield of inherited heart diseases and frequency of cardiac events in SCD relatives in Denmark, from 2005–18. Relatives underwent guideline-recommended screening and follow-up. A total of 686 relatives (47% males, median baseline age 35 years) to 299 probands (75% males, median death age 41 years) were followed for a median of 10.6 years. At 10-year follow-up, 12% of relatives were diagnosed with an inherited heart disease, with the vast majority (93%) diagnosed within 5 years. Cardiac events occurred in 3% and 4% of relatives after 5- and 10-year follow-up, respectively. Overall, 48% of SCD families in the study had an inherited heart disease. The authors conclude that long-term follow-up identified an inherited heart disease in 12% of SCD relatives, primarily diagnosed within 5 years, with rare cardiac events, suggesting that follow-up could be stopped by 5 years for most adult SCD relatives, with guideline-based family screening securing a low occurrence of cardiac events (4%).

This is an important study evaluating the outcomes of clinical screening in the relatives of young SCD victims, rates of diagnosis, and when follow-up can be stopped in those relatives with no evidence of clinical disease. There are very little data in the literature to answer these key questions, and so the data presented in this 10-year follow-up study are invaluable, and come from a prominent, globally recognized team in the SCD field from Denmark.

A critical starting point in determining how a family will be followed up is identification of the precise cause of SCD in the relative. This ideally starts with a comprehensive autopsy in the decedent, performed at a specialist forensic centre with expertise in the evaluation of young SCD.^7–9^ The autopsy investigation should include obtaining key premorbid information, such as previous syncope, family history, results of any prior investigations (e.g. ECG), and the circumstances of the sudden death event. The autopsy investigation should include detailed macroscopic and microscopic evaluation, and a blood sample collected for genetic analysis. In 50–70%, a cause of SCD is established (namely ACM and HCM), and family screening can then be targeted at a particular disease.

Unfortunately, in up to 40% of young SCD cases aged 1–35 years, no cause of death is identified.^1,10^ This makes family screening a major challenge as the screening is not directed at a specific inherited heart disease. Furthermore, the family is left with no answers as to why their young relative died suddenly and is therefore highly anxious about future young SCD cases in the family. Over recent years, the role of genetic testing in the post-mortem blood sample collected from the SCD victim, the so-called ‘molecular autopsy’, has genetically identified a cause of SCD in an additional 15–20% of young SCD cases.^10–13^ These genetic causes include the inherited arrhythmia syndromes such as LQTS and BrS, as well as genetic causes of cardiomyopathies such as HCM and arrhythmogenic right ventricular cardiomyopathy in the absence of structural changes at autopsy, namely the ‘concealed cardiomyopathies’.^14,15^

Both in families where the cause of SCD is established (an inherited heart disease based on autopsy findings and/or genetic cause) and in families where no cause of SCD is found, screening of all first-degree relatives is recommended, especially as most inherited heart diseases are passed on in families as an autosomal dominant trait. This usually includes an ECG, 2D-echocardiogam, Holter monitoring, and an exercise stress test. Additional tests such as cardiac MRI scans, provocation tests, and invasive electrophysiologic studies are adjuvant tests that may be recommended dependent on the particular clinical circumstances. In the current study, over 92% had both an ECG and echocardiogram. If a genetic cause is identified in the family, then cascade genetic testing in the family will accurately determine which relatives carry the genetic cause (and therefore will require long-term follow-up) and which relatives do not carry the genetic cause (and can be released from life-long clinical surveillance). In the current study, 12% of relatives were diagnosed with an inherited heart disease at 10-year follow-up, with the vast majority (93%) diagnosed within 5 years, and cardiac events were rare at 4%. Based on these findings, the authors suggest that follow-up in these SCD families could be stopped by 5 years for most adult SCD relatives, especially if they have no diagnosis, no previous cardiac events, and are not from a family with an identified inherited heart disease.

Making a recommendation of setting a limit of 5 or 10 years of follow-up for these relatives of young SCD cases has its challenges. Certainly, this recommendation should not apply to children, who may develop phenotypic evidence of the condition later in life. For example, a 5-year-old first-degree relative of the SCD victim would likely need 15 years of follow-up in the inherited heart diseases where the phenotype develops in early adulthood. Conversely, this study may support time-limited screening of older at-risk first-degree relatives including the parental generation. A further challenge and more difficult to measure outcome is the ‘reassurance’ a normal clinical screen brings to surviving relatives. Many families develop a long-term relationship with their inherited heart disease service after the death of a loved one, both clinical and research-based, and continue periodic clinical screening over their lifetime even if understanding the expectation of diagnosing a condition is small. These factors need to be considered also if changes in the duration of follow-up in SCD relatives are to be recommended.

**Figure 1:**
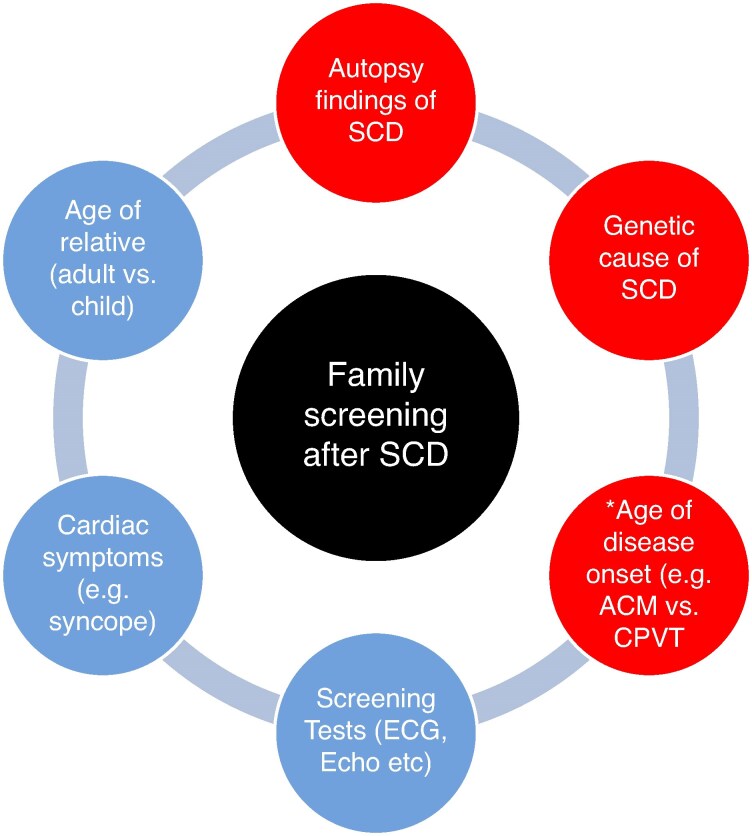
Factors that may influence the frequency and duration of family screening after SCD. Factors related to the cause of SCD based on autopsy and/or genetic findings (in red), and factors related to the family member being screened (in blue). ACM, arrhythmogenic cardiomyopathy; CPVT, catecholaminergic polymorphic ventricular tachycardia. *As an example, CPVT often presents in childhood, while ACM often presents in early adulthood.

What are the implications of the current study for the clinician looking after families after a young SCD? *Figure 1* summarizes the various factors that may influence the frequency and duration of family screening after SCD. Inherited heart diseases are a major cause of SCD in the young. A direct consequence of this is the recommendation that all first-degree relatives of the decedent should be offered clinical screening, as well as genetic testing if a genetic cause has been identified at autopsy.^8^ Based on the current study, a follow-up of 5 years identified 93% of relatives with an inherited heart disease with a low cardiac event rate of 3%. It would therefore not be unreasonable to suggest adult SCD relatives undergo screening for 5 years then cease follow-up, especially if they have no diagnosis, no previous cardiac events, and are not from a family with an identified inherited heart disease. These individuals need to be informed that if they develop new cardiac symptoms in the future, they should undergo basic cardiology review. Importantly, every young SCD family is different, such that duration and frequency of follow-up need to be individualized, especially in children, with the ultimate goal to prevent any future SCD events in the family.
